# A Unique *SLC26A4* Mutation Spectrum in a Mongolian Enlarged Vestibular Aqueduct Cohort via Whole-Exome Sequencing: A Preliminary Study

**DOI:** 10.3390/ijms27125364

**Published:** 2026-06-14

**Authors:** Jargalkhuu Erdenechuluun, Bayasgalan Gombojav, Tserendulam Batsaikhan, Yue-Sheng Lu, Narandalai Danshiitsoodol, Zaya Makhbal, Maralgoo Jargalmaa, Tuvshinbayar Jargalkhuu, Ho-Peng Hsu, Pei-Hsuan Lin, Hung-Ju Su, Chien-Hsing Lin, Yu-Ting Chiang, Chuan-Jen Hsu, Pei-Lung Chen, Jacob Shu-Jui Hsu, Cheng-Yu Tsai, Chen-Chi Wu

**Affiliations:** 1 Department of Otolaryngology, School of Medicine, Mongolian National University of Medical Sciences, Ulaanbaatar 14210, Mongolia; jargalkhuu@mnums.edu.mn; 2 The EMJJ Otolaryngology Hospital, Ulaanbaatar 14210, Mongolia; 3 Department of Epidemiology and Biostatistics, School of Public Health, Mongolian National University of Medical Sciences, Ulaanbaatar 14210, Mongolia; bayasgalan.g@mnums.edu.mn; 4 International Cyber Education Center, Graduate School, Mongolian National University of Medical Sciences, Ulaanbaatar 14210, Mongolia; 5 Department of Otolaryngology, National Taiwan University Hospital, Taipei 100225, Taiwan; 6 Institute of Molecular Medicine, National Taiwan University College of Medicine, Taipei 100225, Taiwan; 7 Department of Probiotic Sciences for Preventive Medicine, Graduate School of Biomedical and Health Sciences, Hiroshima University, Hiroshima 734-8553, Japan; 8 Department of Otolaryngology, National Taiwan University College of Medicine, Taipei 100225, Taiwan; 9 Graduate Institute of Clinical Medicine, National Taiwan University College of Medicine, Taipei 100229, Taiwan; 10 Phalanx Biotech Inc., Hsinchu 302401, Taiwan; 11 Graduate Institute of Medical Genomics and Proteomics, National Taiwan University College of Medicine, Taipei 100233, Taiwan; 12 Department of Otolaryngology, Taichung Tzu Chi Hospital, Taichung 427003, Taiwan; 13 Department of Medical Genetics, National Taiwan University Hospital, Taipei 100226, Taiwan; 14 Department of Medical Research, National Taiwan University Hospital Hsin-Chu Branch, Hsinchu 302041, Taiwan; 15 Department of Otolaryngology, National Taiwan University Hospital Hsin-Chu Branch, Hsinchu 302041, Taiwan

**Keywords:** sensorineural hearing loss, enlarged vestibular aqueducts, cochlear incomplete partition, whole exome sequencing, DFNB4, *SLC26A4*

## Abstract

Enlarged vestibular aqueduct (EVA) is a common inner ear malformation that causes sensorineural hearing loss. It is frequently associated with pathogenic variants in the *SLC26A4* gene. This study aimed to investigate the genetic basis of hearing loss in Mongolian patients with EVA. Whole-exome sequencing was performed in 19 Mongolian patients from 15 unrelated families diagnosed with EVA with or without cochlear incomplete partition type II. All patients underwent high-resolution computed tomography of the temporal bone to confirm the diagnosis. Biallelic *SLC26A4* pathogenic variants were identified in all 15 families, achieving a 100% diagnostic yield. The most frequent variant was c.919-2A>G (40%), followed by c.2027T>A (23.3%) and c.1318A>T (16.7%). The spectrum of variants includes population-specific variants found in East Asians (c.919-2A>G), North Asians (c.2027T>A), and Southwest Asians (c.716T>A), suggesting a unique mutation spectrum in this Mongolian cohort characterized by variants prevalent across various Eurasian populations, which remains to be confirmed in larger studies. Furthermore, correlation analyses on multi-ethnic allele frequencies of biallelic *SLC26A4* genotypes demonstrated positive correlations with deaf cohorts of East Asian, North Asian, Northeast Asian, and Western Asian groups. Digenic inheritance (with pathogenic variants in *FOXI1*, *KCNJ10*, or *EPHA2*) was not observed, and there was no clear genotype–phenotype correlation between specific *SLC26A4* genotypes and hearing levels or inner ear malformations. This study provides a comprehensive overview of the genetic landscape of EVA in the Mongolian population. The identification of biallelic *SLC26A4* pathogenic variants in all families underscores the clinical role of this gene in EVA pathogenesis. The observed pan-ethnic mutation spectrum likely reflects the genetic diversity resulting from historical migrations of Mongolians.

## 1. Introduction

Sensorineural hearing loss (SNHL) affects an estimated 34 million children globally [1], and approximately half of these cases are attributed to genetic factors [2,3]. Recessive variants in the *SLC26A4* gene are the second most common cause of genetic hearing loss worldwide, trailing only *GJB2* variants [4]. The *SLC26A4* gene, located on chromosome 7 [5], encodes the pendrin protein, an anion exchanger crucial for function in the inner ear [6,7,8,9], thyroid, and kidneys [5,10]. Pathogenic variants in *SLC26A4* cause autosomal recessive non-syndromic SNHL, known as DFNB4 (OMIM #600791) [11,12], or Pendred syndrome (PS, OMIM #605646) [13,14,15]. DFNB4 is characterized by inner ear malformations, including enlarged vestibular aqueduct (EVA) and cochlear incomplete partition type II (IP-II). PS presents with similar inner ear malformations, but also includes thyroid goiter with iodine organification defect [12,16,17].

To date, approximately 600 pathogenic or likely pathogenic variants in the *SLC26A4* gene have been documented in the variant databases ClinVar [18] and DVD [19] (last accessed on 10 December 2025). Notably, different ethnic populations harbor distinct *SLC26A4* founder variants and variant landscapes [20,21]. For example, c.1246A>C (p.T416P) and c.1001G>A (p.G334E) are the predominant pathogenic variants in Caucasian populations and c.2168A>G (p.H723R) is prevalent in Japanese and Korean populations, whereas c.919-2A>G is most common in Han Chinese and Taiwanese populations [22,23,24,25,26].

In a previous study in 2018, we applied hot spot mutation screening in a Mongolian cohort of 188 SNHL patients and identified a unique *GJB2* mutation spectrum in Mongolian patients, significantly different from other Eurasian populations [27]. However, comprehensive genetic data for *SLC26A4* was limited; only hotspot mutations could be screened, and a definitive diagnosis of EVA was often hindered by the lack of temporal bone imaging. Therefore, this study aims to re-examine the genetic landscape and mutational spectrum of *SLC26A4* variants in a Mongolian cohort confirmed with EVA on imaging studies using whole-exome sequencing (WES). Specifically, we sought to characterize the genetic profile of this underrepresented population to test the hypothesis of a unique pan-ethnic *SLC26A4* spectrum reflecting historical migrations and complex East–West Eurasian genetic interactions.

## 2. Results

### 2.1. Categories of Clinical Phenotypes in Mongolian EVA Patient Cohort

The study cohort consisted of 19 patients from 15 unrelated families. Detailed individual audiological parameters, including symmetry and age of diagnosis, are provided in Table 1. The mean age of the cohort was 8.2 ± 5.1 years, with ages ranging from 2 to 19 years old. The gender distribution was 8 males (42.1%) and 11 females (57.9%). Four children (21.1%) presented with EVA alone (normal cochlear partition), while 15 (78.9%) had IP-II additionally. All participants had bilateral SNHL, with five children (26.3%) having severe SNHL and fourteen children (73.7%) having profound SNHL. Table 2 summarizes the statistical data and clinical characteristics of the cohort. There were no statistically significant differences in age, hearing level, or sex between the EVA-alone and EVA-with-IP-II groups (*p* = 0.414, 0.721, and 0.941, respectively). Regarding potential syndromic manifestations associated with *SLC26A4* variants, none of the patients in this cohort presented with palpable goiter or clinical evidence of thyroid dysfunction at the time of evaluation (Table 1). Given that individuals with Pendred syndrome typically develop goiter during or after adolescence, the absence of this feature in our patients may be attributed to their young age. This finding is consistent with previous reports indicating that pediatric patients with *SLC26A4*-related hearing loss frequently maintain euthyroid status during early childhood.

### 2.2. Identification of Biallelic SLC26A4 Variants

All probands of the 15 unrelated Mongolian families were confirmed to carry biallelic *SLC26A4* pathogenic variants, resulting in a 100% diagnostic yield (Table 3), including 11 families (Family ID 1 to 11) reported in our previous study [28] and four families (Family 12 to 15) newly added in this study. These variants were found in either homozygous or compound heterozygous states. Seven patients segregated homozygous variants: three with c.919-2A>G (MG0295 and MG0499 of Family 1 and MG0572 of Family 11), three with c.1318A>T (MG0711, MG0712, and MG0713 of Family 13), and one with c.2027T>A (MG0309 of Family 6). The remaining 12 patients segregated compound heterozygous *SLC26A4* variants.

**Figure 1:**
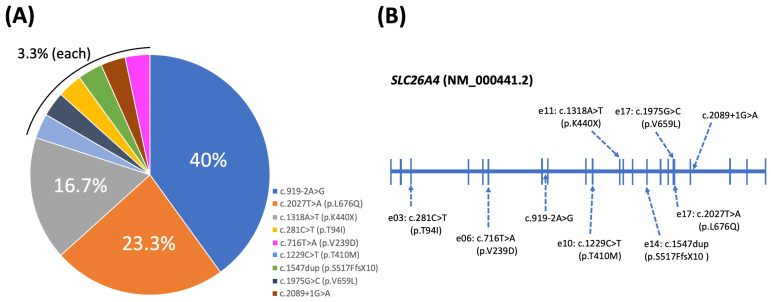
Summary of the Mongolian *SLC26A4* mutational spectrum across the 15-family study cohort. (**A**) Statistical distribution of *SLC26A4* variants across 15 unrelated families (total of 30 alleles). The three most common variants—c.919-2A>G (40%), c.2027T>A (23.3%), and c.1318A>T (16.7%)—constitute the vast majority of the landscape, followed by six sporadic variants (each representing 3.3% of alleles). (**B**) Schematic diagram mapping all nine identified variants across the *SLC26A4* transcript (NM_000441.2). The horizontal backbone represents the intronic regions, while the vertical bars delineate individual exons (with “e” numbering corresponding to specific exon locations), illustrating the distribution of missense, nonsense, duplicating, and splice-site variants across the gene structure.

As summarized in Figure 1A, a total of nine *SLC26A4* variants were identified in the cohort; the most frequent variant was c.919-2A>G, found in 12 out of 30 alleles (40%). The second most common variant was c.2027T>A (p.L676Q), found in 7 out of 30 alleles (23.3%). The third most common was c.1318A>T (p.K440X), accounting for 5 out of 30 alleles (16.7%). Other variants identified sporadically included c.281C>T (p.T94I), c.716T>A (p.V239D), c.1229C>T (p.T410M), c.1547dup (p.S517FfsX10), c.1975G>C (p.V659L), and c.2089+1G>A, collectively constituting the Mongolian *SLC26A4* mutational spectrum in this study (Figure 1B). The genotype–phenotype relationship integrating all *SLC26A4* genotypes with hearing levels and imaging features presented in Table 3, along with the pathogenicity assessments and corresponding details of each *SLC26A4* variant, are listed in Table 4. No pathogenic or likely pathogenic variants were identified in other known deafness-associated genes or classical EVA-associated genes (*FOXI1*, *KCNJ10*, and *EPHA2*), supporting a non-digenic, purely *SLC26A4*-driven etiology in this cohort.

### 2.3. Genotype–Phenotype Correlation and Progression

No clear correlation was observed between specific *SLC26A4* genotypes and the level of hearing loss. To statistically evaluate this, mean hearing levels (PTA) were compared across different variant groups (homozygotes vs. compound heterozygotes), revealing no statistically significant differences (*p* > 0.05, Kruskal–Wallis test). Individuals with the same genotype, such as c.919-2A>G homozygotes (MG0295, MG0499, and MG0572), demonstrated variable phenotypes, presenting with either profound or severe hearing loss. Notably, two boys, aged 11 (MG0572, *SLC26A4*: c.[919-2A>G];[919-2A>G]) and 7 (MG0626, *SLC26A4*: c.[c.1975G>C];[2027T>A]), showed an initial diagnosis of normal hearing followed by subsequent deterioration, suggesting factors beyond the *SLC26A4* genotype may contribute to the variability in hearing loss progression. Similarly, no clear correlation was found between specific *SLC26A4* genotypes and inner ear malformations (EVA alone vs. EVA with IP-II).

### 2.4. Pan-Ethnic Mutational Spectrum Analysis

The identified *SLC26A4* variants demonstrated a pan-ethnic distribution, incorporating variants prevalent in geographically distant populations. This spectrum included c.919-2A>G (common in East Asians), c.2027T>A (prevalent in North Asians), and c.716T>A (prevalent in Southwest Asians). We also summarized the previous studies of clinical genetics across a total of six deaf populations (four population-prevalent variants in Table 5 and all variants in Table S1), in terms of the Mongolian *SLC26A4* variants, to comprehend their proportion of *SLC26A4*-related etiology, and the corresponding allele frequencies in each cohort. Multiple deaf populations demonstrated various proportions of *SLC26A4*-related etiology, ranging from 5.67 (Northeast Asian) to 21.09% (North Asian) in biallelic genotype. Statistical correlation analysis of biallelic *SLC26A4* allele frequencies between the Mongolian deaf cohort and six multi-ethnic deaf cohorts revealed positive Pearson correlations (*r*) with four major groups with *p* < 0.05 (Table 6): North Asian (*r* = 0.920), East Asian (*r* = 0.828), Northeast Asian (*r* = 0.811), and Western Asian (*r* = 0.678).

## 3. Discussion

In this study, we provide a comprehensive summary of the genetic etiologies in Mongolian patients with EVA, with or without cochlear IP-II. By employing a WES approach, we successfully identified biallelic *SLC26A4* pathogenic variants in all families.

### 3.1. Unique Mutational Spectrum and Diagnostic Yield

The most prevalent variant in our cohort was c.919-2A>G (40%), followed by c.2027T>A (p.L676Q, 23.3%), and c.1318A>T (p.K440X, 16.7%). We identified a total of nine *SLC26A4* variants, representing a broader spectrum than previously found using hotspot screening methods [27]. This demonstrates the enhanced medical and academic utility of WES in clinical diagnostics for the precision medicine [53].

Our study achieved a high diagnostic yield (100%) for biallelic *SLC26A4* variants in this specific cohort of Mongolian patients with EVA. This yield is substantially higher than figures reported in other populations, specifically in East Asian studies (approximately 80% in Japanese and Korean populations) [24,25,54] and significantly higher than in Caucasian studies (approximately 25% in European and North American populations) [55,56]. This high yield is attributable to the strong association between *SLC26A4* variants and the rigorously defined clinical phenotypes of EVA and IP-II [16], coupled with the comprehensive variant detection capability of the WES approach. However, it should be noted that this 100% yield is likely a reflection of our small sample size and rigorous inclusion criteria (requiring radiologically confirmed EVA and IP-II), and it may not represent the diagnostic yield in a more generalized Mongolian pediatric deaf population.

### 3.2. Absence of Genotype–Phenotype Correlation and Digenic Inheritance

Consistent with other studies, we did not observe a clear correlation between specific *SLC26A4* genotypes and hearing features or inner ear malformations in our cohort. While some literature has reported certain *SLC26A4* genotypes to be associated with less severe hearing loss [57,58], more studies have suggested that there is no clear genotype–phenotype correlation [37,44,59]. This lack of correlation may be attributed to the allelic heterogeneity of *SLC26A4*, where the effect of numerous variants on protein function varies. In addition, environmental factors, such as head trauma, can influence hearing loss progression in individuals with *SLC26A4* variants [60,61]. While head trauma has been noted as a potential factor in *SLC26A4*-related hearing deterioration, our cohort showed variable progression that did not always correlate with reported trauma, as detailed in Table 1. Notably, while European cohorts often identify diverse genetic etiologies—such as *EYA1* variants or the CEVA haplotype—in broader EVA populations [62], our 100% diagnostic yield underscores the high specificity of utilizing strict radiological criteria (>2.0 mm midpoint) to capture *SLC26A4*-specific phenotypes.

Digenic inheritance, which occurs when a mono-allelic *SLC26A4* variant is combined with another variant in genes such as *FOXI1* [63], *KCNJ10* [64], or *EPHA2* [65], has been proposed as a possible cause of DFNB4 and PS. However, we did not find any cases of digenic inheritance in our cohort. All patients with confirmed genetic etiology had biallelic *SLC26A4* mutations, and no pathogenic or likely pathogenic variants were identified in *FOXI1*, *KCNJ10*, or *EPHA2*. These results suggest that digenic inheritance may play a less significant role in the Mongolian population than in other populations.

### 3.3. Pan-Ethnic Genetic Landscape of Mongolian SLC26A4 Variants

Our Mongolian cohort exhibits a pan-ethnic mutation spectrum, consistent with previous reports on Mongolian genetic profiles [20,27]. This is the most significant finding of our study, demonstrating a genetic signature influenced by diverse Eurasian ancestral populations. The spectrum includes variants prevalent in distinct ethnic groups, including the most common c.919-2A>G variant, which is widespread in East Asian populations [14,32,41,43,44,45,46,66,67]; the c.2027T>A (p.L676Q) variant, which is prevalent in North Asian groups, such as Tuvinian and Altai patients [43]; and the c.716T>A (p.V239D) variant, which is prevalent in Southwest Asians, such as Iranians, Indians Pakistanis [14,36,46]. We also detected c.1547dup (p.S517FfsX10), prevalent in Thai patients [66]. Interestingly, the geographic distribution of *SLC26A4* pathogenic variants within this Mongolian EVA cohort (Figure S1) revealed that the c.919-2A>G variant was predominant across the entire Mongolian population. In contrast, other major variants exhibited distinct regional clustering: c.2027T>A (p.L676Q), c.1318A>T (p.K440X), and c.716T>A (p.V239D) were predominantly identified in the northern, eastern, and southern regions of Mongolia, respectively. This localized stratification may imply a positive correlation between their geographic distribution and previously reported ancestral population dynamics.

We hypothesize that this unique landscape may be a consequence of the complex genetic transmission of ancestral Mongolian genomes, shaped by geographical factors, political history, and the migration and interaction of various ethnic groups throughout history [68,69,70]. However, this interpretation remains preliminary given our sample size. Previous cluster analyses have noted genetic similarity between the Mongolian *SLC26A4* mutational spectrum and those of East Asian, Northeast Asian, and South Asian populations [20]. A comprehensive genetic study by Bai et al. [71] showed that Mongolians have a close phylogenetic relationship with Siberians (North Asian), East Asians, and Southeast Asians.

To delineate this relationship, we also conducted statistical analyses based on the allele frequencies of biallelic *SLC26A4* variants in multi-ethnic deaf cohorts (Table 5 and Table S1), demonstrating significant positive Pearson correlations with multiple populations (Table 6): North Asian (*r* = 0.920), East Asian (*r* = 0.828), Northeast Asian (*r* = 0.811), and Western Asian (*r* = 0.678) with *p* < 0.05. These findings support the conclusion of a shared mutational spectrum [20] and a closer phylogenetic relationship among North, East, and Northeast Asian populations [71].

Notably, in this study the nonsense variant c.1318A>T (p.K440X) was captured in two of these new families, presenting in a homozygous state in Family 13 and a compound heterozygous state in Family 15. Additionally, the splicing variant c.2089+1G>A was identified in Family 14. As detailed in Table 6 and Table S1, both variants are rarely reported in the East Asian cohort, and identifying them in these new families effectively expands the known *SLC26A4* disease-causing spectrum to nine distinct variants within this specific cohort. These findings were only possible through the expanded dataset and comprehensive WES assays, thereby solidifying the cross-population genetic proximity across these geographic regions.

Based on previous genetic and ancestral studies [70,71] and our multiethnic analyses, the Mongolian mutational spectrum is likely influenced by: (1) geographical distance from other ethnic groups, (2) ethnic-specific ancestral proportions in the Mongolian genomic background, and (3) allelic frequencies of common *SLC26A4* variants in each ethnic group. This hypothesis requires validation with more comprehensive genomic databases of Eurasian populations and a larger Mongolian cohort of EVA.

Furthermore, it is worth noting that the population datasets compared in Table 5 and Table 6 originate from diverse historical studies with varying diagnostic frameworks and patient inclusion criteria. While these cohorts provide a valuable baseline for comparing SNV spectrums across regions, a critical limitation is the technical inability of conventional sequencing methods (e.g., Sanger sequencing or short-read NGS assays) to accurately resolve copy number variations (CNVs). The allelic frequencies of pathogenic variants in these population datasets may be underestimated. Therefore, more emerging datasets integrating advanced workflows, such as long-read sequencing, are needed to enable more precise multi-group correlation analyses in the future.

To our knowledge, this study is the first detailed investigation into the genetic landscape of EVA in the Mongolian population, integrating the comprehensive audiological characterization of a 15-family cohort with WES data to hypothesize a unique pan-ethnic mutational spectrum influenced by Eurasian migrations. However, our study is limited by its small sample size. This limitation may have prevented the delineation of robust genotype–phenotype correlations and the detection of additional rare variants contributing to EVA. Notably, the variant c.2168A>G (p.H723R), commonly found in Japanese [24,44] and Korean deaf populations [25,45,54], was absent in our cohort. This absence may be attributed to the fact that our sampling was entirely from Mongolia (Outer Mongolia, see Table 3 and Figure S1). This contrasts with previous studies that included populations from Inner Mongolia [14,30]. Inner Mongolian populations historically have had greater interaction with Northeast Asian groups [72] and a different immigration history [73], which may explain the higher prevalence of c.2168A>G carriers in those regions.

## 4. Materials and Methods

### 4.1. Subjects

Nineteen Mongolian patients from 15 unrelated families were recruited between 2022 and 2024. This includes eleven families (Family ID 1 to 11) reported in our previous WES study [28], along with four families (Family ID 12 to 15) that were newly recruited in 2024 WES assays newly reported in this study. All patients were diagnosed with EVA with or without cochlear IP-II, as confirmed by high-resolution computed tomography (HRCT) imaging of the temporal bone. Following the criteria established in our previous studies, EVA was defined as a vestibular aqueduct midpoint width exceeding 2.0 mm on axial HRCT, while IP-II was identified by a cystic cochlear apex with a normal basal turn [26,59]. All HRCT images were independently evaluated by two pediatric otologists to ensure diagnostic precision. These rigorous imaging evaluations are critical for establishing genotype–phenotype correlations, as highlighted by Bałdyga et al. [62]. All participants provided written informed consent for genetic testing before undergoing WES. This study was approved by the research ethics committees of the National Taiwan University Hospital (NTUH) (202007065RINB) and the EMJJ Otolaryngology Hospital of Mongolia (Medical Ethics Committee of the Ministry of Health, Mongolia; No: 268 & 23/065).

### 4.2. Clinical Examinations

Detailed clinical data were collected from the proband and affected members of each family, including a comprehensive family history, personal medical history, and physical examination. Audiological evaluations were performed using pure-tone audiograms or auditory brainstem response, depending on the age or neurological status of the patient. Hearing levels were determined by averaging the air conduction thresholds at 0.5, 1, 2, and 4 kHz from both ears. Hearing levels of the better ears were classified according to WHO guidelines as mild (26–40 decibel hearing level [dBHL)), moderate (41–60 dBHL), severe (61–80 dBHL), or profound (>81 dBHL) [74]. HRCT of the temporal bone was performed using 0.6 mm contiguous axial and coronal sections to evaluate the structure of the inner ear [75].

### 4.3. Genetic Sequencing and Analysis

Genomic DNA was extracted from dried blood spot samples from each participant. WES was performed using the NovaSeq 6000 platform (Illumina Inc., San Diego, CA, USA). As reported in our previous study [28], the WES workflow achieved a sequencing depth of >30x for at least 90% of the targeted regions. Raw reads were aligned to the human reference genome (hg38) using BWA (version 0.7.17) [76]. Variant calling and genotyping were performed via GATK (version 3.4) [77], followed by functional annotation using ANNOVAR (version 2016) [78]. Validation of the identified *SLC26A4* variants was performed using Sanger sequencing. To establish the meiotic phase of the compound heterozygous variants, parental segregation analysis was systematically conducted wherever parental DNA samples were accessible, confirming that these variants were inherited in trans within the verified families. The identification of causative variants was based on several factors: minor allele frequency (MAF) of less than 1% in the gnomAD population database (version 4.1) [40], correlation with patient phenotypes and medical records, and evidence from disease databases such as ClinVar (last accessed on 19 May 2026) [18] and the Deafness Variation Database (DVD) (version 9) [19]. Pathogenicity was assessed using a variety of prediction tools, including CADD (version 1.4) for all variant types; Sorting Intolerant From Tolerant (SIFT) (version 2019) [79] and Polymorphism Phenotyping (PolyPhen-2) (version 2) [80] for missense variants; and SpliceAI (version 1.3) [81] for intronic variants. Pathogenicity classification was performed according to the American College of Medical Genetics and Genomics (ACMG) guidelines [82] and its specified rule for hearing loss [83]. To evaluate potential digenic inheritance patterns, the WES dataset was systematically filtered to screen all known hereditary hearing loss genes, focusing specifically on *FOXI1* [63], *KCNJ10* [64], or *EPHA2* [65], which have been previously implicated in digenic enlargement of the vestibular aqueduct. The depth-of-coverage computational algorithms DECoN (version 2.0) [84] and CODEX2 (version 1.3.0) [85] were also utilized to detect potential copy number variations (CNVs) from the WES data. Genomic profiles from healthy controls were utilized as reference controls for read-depth normalization across target exons. No positive or clinically relevant pathogenic CNVs were identified within the *SLC26A4* region in this Mongolian cohort.

### 4.4. Statistical Analysis

Continuous variables are presented as mean ± standard deviation (SD) and were compared using the Wilcoxon rank-sum test to analyze differences between independent clinical groups (EVA alone vs. EVA with IP-II). For multigroup comparisons of hearing thresholds across distinct genotype groups, the Kruskal–Wallis test was applied. Categorical variables are presented as numbers and percentages and were analyzed using Fisher’s exact test for all contingency table evaluations. To assess the similarity of the mutational landscape between the Mongolian cohort and multi-ethnic populations, cross-population allele frequency datasets were evaluated using the Pearson correlation coefficient (*r*), with corresponding *p*-values and 95% confidence intervals (CIs) calculated. Statistical significance was defined as a *p*-value < 0.05. All statistical analyses were performed using R version 4.4.1 (Race for Your Life).

## 5. Conclusions

This study, utilizing WES on a rigorously defined cohort of patients with EVA, provides a comprehensive overview of the genetic landscape of EVA in the Mongolian population. Remarkably, we achieved a 100% diagnostic yield for biallelic *SLC26A4* pathogenic variants in all families, underscoring the clinical role of this gene in EVA pathogenesis within this ethnic group. In this cohort, the *SLC26A4* mutational spectrum appears to incorporate variants common to diverse East, North, and Southwest Asian populations. While these findings may reflect the genetic diversity resulting from historical migrations, further studies with larger cohorts are strictly necessary to confirm these patterns and establish a robust genetic profile for the broader Mongolian population. Consistent with many international studies, we found no clear genotype–phenotype correlations within this specific cohort or evidence of digenic inheritance in EVA-related genes (*FOXI1*, *KCNJ10*, or *EPHA2*), though our small sample size may be statistically underpowered to preclude an underlying biological correlation. Identifying this pan-ethnic spectrum provides an effective basis for genetic screening in the Mongolian population. Further studies with larger cohorts are needed to elucidate the complete genetic complexity of EVA in this population and establish robust genotype–phenotype relationships.

## Figures and Tables

**Table 1:** Demographic and clinical characteristics of the 19 Mongolian patients with EVA.

Family ID	Sample ID	Age/ Gender	PTA for Each Ear (L: Left; R: Right)	Symmetry	Progression	ABR/OAE	Head Trauma	Hearing Aid (HA) or Cochlear Implant (CI)	HRCT	Goiter	Thyroid Dysfunction
1-I	MG0295	16/F	R-93 dB/L-90 dB	Symmetrical	Stable	ABR, OAE absent	Nil	Right HA (left without hearing assistive device)	Bilateral EVA only	Nil	Nil
1-II	MG0499	13/M	R-76 dB/L-85 dB	Symmetrical	Stable	ABR (wave V: R-90 dB, L-100 dB) OAE absent	Nil	Right HA (left without hearing assistive device)	Bilateral EVA only	Nil	Nil
2	MG0370	11/M	R-88 dB/L-66 dB	Symmetrical	Stable	ABR (wave V: R-100 dB, L-80 dB) OAE absent	Nil	Left HA (right without hearing assistive device)	Bilateral EVA only	Nil	Nil
3	MG0338	4/M	R-50 dB/L-83 dB	Symmetrical	Progressive	ABR not tested OAE absent	Nil	Bil HA	Bilateral EVA with IP-II	Nil	Nil
4-I	MG0348	7/F	R-100 dB/L-91 dB	Symmetrical	Stable	ABR, OAE absent	Nil	Right CI and Left HA	Bilateral EVA with IP-II	Nil	Nil
4-II	MG0349	8/F	R-100 dB/L-100 dB	Symmetrical	Stable	ABR (wave V: R-absent, L-100 dB) OAE absent	Nil	Left HA (right without hearing assistive device)	Bilateral EVA with IP-II	Nil	Nil
5	MG0293	5/F	R-88 dB/L-78 dB	Symmetrical	Stable	ABR (wave V: R-100 dB, L-100 dB) OAE absent	Nil	Bil HA	Bilateral EVA with IP-II	Nil	Nil
6	MG0309	3/M	R-90 dB/L-66 dB	Symmetrical	Stable	ABR not tested OAE absent	Nil	Bil HA	Bilateral EVA with IP-II	Nil	Nil
7	MG0389	4/F	R-65 dB/L-71 dB	Symmetrical	Progressive	ABR (wave V: R-90 dB, L-90 dB) OAE absent	Fell off the bed in 2018	Bil HA	Bilateral EVA with IP-II	Nil	Nil
8	MG0419	2/F	R-88 dB/L-90 dB	Symmetrical	Stable	ABR (wave V: R-100 dB, L-100 dB) OAE absent	Nil	Bil CI	Bilateral EVA with IP-II	Nil	Nil
9	MG0527	14/F	R-91 dB/L-85 dB	Symmetrical	Stable	ABR (wave V: R-absent, L-100 dB) OAE absent	Nil	Left CI (right without hearing assistive device)	Bilateral EVA with IP-II	Nil	Nil
10	MG0572	11/M	R-71 dB/L-51 dB	Symmetrical	Stable	ABR not tested OAE absent	Nil	Bil HA	Bilateral EVA with IP-II	Nil	Nil
11	MG0626	7/M	R-86 dB/L-73 dB	Symmetrical	Stable	ABR (wave V: R-90 dB, L-80 dB) OAE absent	Nil	Bil HA	Bilateral EVA with IP-II	Nil	Nil
12	MG0496	2/F	R-100 dB/L-91 dB	Symmetrical	Stable	ABR (wave V: R-absent, L-90 dB) OAE absent	Nil	Bil HA	Bilateral EVA with IP-II -	Nil	Nil
13-I	MG0711	13/F	R-120 dB/L-83 dB	Symmetrical	Stable	ABR (wave V: R-absent, L-90 dB) OAE absent	Nil	Left HA (right without hearing assistive device)	Bilateral EVA with IP-II	Nil	Nil
13-II	MG0712	19/F	R-113 dB/L-110 dB	Symmetrical	Progressive	ABR, OAE -not tested	Nil	Right HA (left without hearing assistive device)	Bilateral EVA with IP-II	Nil	Nil
13-III	MG0713	6/M	R-71 dB/L-53 dB	Symmetrical	Progressive	ABR (wave V: R-100 dB, L-70 dB) OAE absent	Nil	Bil HA	Bilateral EVA with IP-II	Nil	Nil
14	MG0676	8/M	R-61 dB/L-71 dB	Symmetrical	Progressive	ABR (wave V: R-90 dB, L-70 dB) OAE absent	Nil	Bil HA	Bilateral EVA with IP-II	Nil	Nil
15	MG0682	2/F	R-88 dB/L-90 dB	Symmetrical	Stable	ABR (wave V: R-100 dB, L-100 dB) OAE absent	Nil	Bil CI	Bilateral EVA with IP-II	Nil	Nil

Abbreviations: ABR, Auditory Brainstem Response; OAE, Otoacoustic Emission; HA, Hearing Aid; CI, cochlear implant; Bil, Bilateral; HRCT, High-Resolution Computed Tomography; EVA, enlarged vestibular aqueduct; IP-II, cochlear incomplete partition type II.

**Table 2:** Summary of demographic and clinical parameters of the Mongolian EVA cohort.

Variables	EVA Alone (*n* = 4)	EVA with IP-II (*n* = 15)	Total (*n* = 19)	*p*-Value
Age (year, mean ± SD)	13.4 ± 6.0	7.5 ± 4.9	8.2 ± 5.1	0.414
Hearing level (dBHL, mean ± SD)	87.6 ± 6.6	89.4 ±14.9	89.0 ± 13.4	0.721
Age of diagnosis	11.3 ± 5.0	5.8 ± 5.4	6.8 ± 5.5	
PTA for right ear	85.7 ± 8.7	72.1 ± 34.4	74.38 ± 31.8	0.518
PTA for left ear	80.3 ± 12.6	79.7 ± 16.1	79. 8 ± 15.2	0.526
ABR right	95.0 ± 7.1	95.7 ± 5.3	95.5 ± 5.2	0.439
ABR left	90.0 ± 14.1	90.0 ± 12.5	90.0 ± 12.1	0.999
Sex				
Male [*n* (%)]	2 (50.0)	6 (40.0)	8 (42.1)	0.941
Female [*n* (%)]	2 (50.0)	9 (60.0)	11 (57.9)	
Age of onset				
Congenital [*n* (%)]	3 (75.0)	13 (86.7)	16 (83.3)	0.396
Acquired [*n* (%)]	1 (25.0)	2 (13.3)	3 (16.7)	
Progression				
Static [*n* (%)]	4 (100.0)	10 (66.7)	14 (72.2)	0.511
Progressive [*n* (%)]	0 (0.0)	5 (33.3)	5 (27.8)	
Hearing aid				
Right [*n* (%)]	2 (50.0)	4 (26.7)	6 (33.3)	0.614
Left [*n* (%)]	1 (25.0)	2 (13.3)	3 (16.7)	
Both [*n* (%)]	1 (25.0)	9 (60.0)	9 (50.0)	

Abbreviations: SD, standard deviation; PTA, Pure-tone audiometry; EVA, enlarged vestibular aqueduct; IP-II, cochlear incomplete partition type II.

**Table 3:** *SLC26A4* genotypes, hearing levels, and inner ear malformation features of the 19 Mongolian patients.

Family ID	Sample ID	Variant in Allele 1		Variant in Allele 2		Hearing Loss Level	Inner ear Malformation	Birthplace	Case Reported ‡
		HGVS (cDNA) #	HGVS (Protein) #	HGVS (cDNA)	HGVS (Protein)				
1-I	MG0295	c.919-2A>G	-	c.919-2A>G	-	profound	EVA alone	Bayan-Ölgii	Reported
1-II	MG0499	c.919-2A>G	-	c.919-2A>G	-	profound	EVA alone	Bayan-Ölgii	Reported
2	MG0370	c.919-2A>G	-	c.281C>T	p.T94I	severe	EVA alone	Ulaanbaatar	Reported
3	MG0338	c.919-2A>G	-	c.2027T>A	p.L676Q	profound	EVA alone	Khentii	Reported
4-I	MG0348	c.1318A>T	p.K440X	c.1229C>T	p.T410M	profound	EVA with IP-II	Ulaanbaatar	Reported
4-II	MG0349	c.1318A>T	p.K440X	c.1229C>T	p.T410M	profound	EVA with IP-II	Ulaanbaatar	Reported
5	MG0293	c.919-2A>G	-	c.716T>A	p.V239D	profound	EVA with IP-II	Bayankhongor	Reported
6	MG0309	c.2027T>A	p.L676Q	c.2027T>A	p.L676Q	profound	EVA with IP-II	Ulaanbaatar	Reported
7	MG0389	c.919-2A>G	-	c.1547dup	p.S517FfsX10	severe	EVA with IP-II	Ulaanbaatar	Reported
8	MG0419	c.919-2A>G	-	c.1318A>T	p.K440X	profound	EVA with IP-II	Dornogovi	Reported
9	MG0527	c.919-2A>G	-	c.2027T>A	p.L676Q	profound	EVA with IP-II	Selenge	Reported
10	MG0572	c.919-2A>G	-	c.919-2A>G	-	severe	EVA with IP-II	Ulaanbaatar	Reported
11	MG0626	c.1975G>C	p.V659L	c.2027T>A	p.L676Q	profound	EVA with IP-II	Ulaanbaatar	Reported
12	MG0496	c.919-2A>G	-	c.2027T>A	p.L676Q	severe	EVA with IP-II	Ulaanbaatar	This study
13-I	MG0711	c.1318A>T	p.K440X	c.1318A>T	p.K440X	profound	EVA with IP-II	Ulaanbaatar	This study
13-II	MG0712	c.1318A>T	p.K440X	c.1318A>T	p.K440X	profound	EVA with IP-II	Ulaanbaatar	This study
13-III	MG0713	c.1318A>T	p.K440X	c.1318A>T	p.K440X	severe	EVA with IP-II	Ulaanbaatar	This study
14	MG0676	c.2027T>A	p.L676Q	c.2089+1G>A	-	profound	EVA with IP-II	Ulaanbaatar	This study
15	MG0682	c.919-2A>G	-	c.1318A>T	p.K440X	profound	EVA with IP-II	Dornogovi	This study

Abbreviations: EVA, enlarged vestibular aqueduct; IP-II, cochlear incomplete partition type II. # HGVS (Human Genome Variation Society) Nomenclature was adopted for the variant descriptions [29]. (ver. 21.1.3 updated on 6 April 2025). ‡ There were 11 unrelated families reported in our previous study [28].

**Table 4:** Pathogenicity assessments and details of Mongolian *SLC26A4* variants in this study.

HGVS (NM_000441.2) & Loci of *SLC26A4* Variants	Grpmax-AF § (Population)	Database Assertions	Prediction Scores #	ACMG Criteria (Classification)	Ref
c.919-2A>G (p.?) *hg38: chr7-107683453-A-G*	0.002630 (EAS)	ClinVar: P DVD: P	SIFT: N.A. PL-2: N.A. CADD: 28.4 DS-AL: 1.0	PVS1, PS4, PP4 (Pathogenic)	[24,30,31,32]
c.2027T>A (p.L676Q) *hg38: chr7-107702050-T-A*	0.00002231 (EAS)	ClinVar: P/LP DVD: LP	SIFT: D PL-2: D CADD: 27.5	PS3, PM2, PM3, PP3, PP4 (Pathogenic)	[14,31,33]
c.1318A>T (p.K440X) *hg38: chr7-107694457-A-T*	N.A.	ClinVar: P/LP DVD: P	SIFT: N.A. PL-2: N.A. CADD (40)	PVS1, PM2, PP4 (Pathogenic)	[31]
c.1229C>T (p.T410M) *hg38: chr7-107690203-C-T*	0.0006687 (EAS)	ClinVar: P DVD: P	SIFT: D PL-2: D CADD (31)	PS3, PM2_P, PM3, PP3, PP4 (Pathogenic)	[30,34,35]
c.281C>T (p.T94I) *hg38: chr7-107663412-C-T*	0.00004457 (EAS)	ClinVar: P DVD: P	SIFT: D PL-2: D CADD: 26.7	PM2, PM3, PP3, PP4 (Likely Pathogenic)	[31]
c.716T>A (p.V239D) *hg38: chr7-107675060-T-A*	0.001570 (SA)	ClinVar: P DVD: P	SIFT: D PL-2: P CADD: 27.6	PS3, PM3, PP3, PP4 (Likely Pathogenic)	[14,35,36]
c.1547dup (p.S517FfsX10) *hg38: chr7-107698042-T-TC*	0.0001339 (EAS)	ClinVar: P/LP DVD: P	SIFT: N.A. PL-2: N.A. CADD: 34	PVS1, PM2_P, PP4 (Pathogenic)	[14,37]
c.1975G>C (p.V659L) *hg38: chr7-107701998-G-C*	0.0002229 (EAS)	ClinVar: P DVD: P	SIFT: D POL2: B CADD: 23.6	PS3, PM2_P, PM3, PP3, PP4 (Pathogenic)	[30,31,35]
c.2089+1G>A (p.?) *hg38: chr7-107704386-G-A*	0.00007351 (AFA)	ClinVar: P DVD: P	SIFT: N.A. PL-2: N.A. CADD: 35 DS-DL: 0.99	PVS1, PM2_P, PP4 (Pathogenic)	[38,39]

Abbreviations: ACMG, American College of Medical Genetics and Genomics; PVS (1), Pathogenic Very Strong; PS (1–4), Pathogenic Strong; PM (1–6), Pathogenic Moderate; PM2_P, PM2_Supporting; PP (1–5), Pathogenic Supporting; EAS, East Asian; SA, South Asian; AFA, African/African American. § Grpmax-AF: The maximum allele frequency across populations in the gnomAD database [40] (ver. 4.1, last accessed on 19 May 2026). # PL-2: PolyPhen-2 (HVAR). Abbreviations of SIFT/PL-2 assertions: D (damaging); P (possibly damaging); B: (benign). Representative maximal SpliceAI scores on targeted variant: DS-AL (Delta Score of Acceptor Loss); DS-DL: (Delta Score of Donor Loss).

**Table 5:** Allelic frequencies of identified *SLC26A4* variants across multi-ethnic cohorts.

Population (Case Number)	Cases in Any Genotype (Case Percent)	Cases in Biallelic Genotype (Case Percent)	Ref	The Allelic Counts (Allelic Frequency) of Each *SLC26A4* Variant in Biallelic Genotype			
				c.919-2A>G	c.2027T>A (p.L676Q)	c.1229C>T (p.T410M)	c.716T>A (p.V239D)
East Asian							
Taiwanese (5184)	346 (6.67)	300 (5.79)	[32]	473 (78.83)	0 (0)	17 (2.83)	0 (0)
Chinese (3379)	654 (19.35)	522 (15.45)	[41,42]	652 (62.45)	25 (2.39)	24 (2.3)	1 (0.1)
Total (8563)	1000 (11.68)	822 (9.6)		1125 (68.43)	25 (1.52)	41 (2.49)	1 (0.06)
North Asian							
Tuvinian (220)	75 (34.09)	62 (28.20)	[43]	86 (69.35)	23 (18.55)	0 (0)	0 (0)
Altaian (93)	5 (5.38)	4 (4.30)	[43]	2 (25)	2 (25)	0 (0)	0 (0)
Total (313)	80 (25.56)	66 (21.09)		88 (66.67)	25 (18.94)	0 (0)	0 (0)
Northeast Asian							
Japanese (1511)	100 (6.62)	66 (4.37)	[44]	13 (9.85)	0 (0)	3 (2.27)	0 (0)
Korean (323)	63 (19.50)	38 (11.76)	[14,45]	22 (28.95)	2 (2.63)	2 (2.63)	0 (0)
Total (1834)	163 (8.89)	104 (5.67)		35 (16.83)	2 (0.96)	5 (2.40)	0 (0)
Western Asian							
Iranian (831)	76 (9.15)	74 (8.9)	[46]	8 (5.41)	2 (1.35)	2 (1.35)	6 (4.05)
Turkey (133)	17 (12.78)	17 (12.78)	[47,48,49]	4 (11.76)	0 (0)	2 (5.88)	0 (0)
Total (964)	93 (9.65)	91 (9.44)		12 (6.59)	2 (1.10)	4 (2.20)	6 (3.30)
South Asian							
Indian (106)	7 (6.60)	7 (6.60)	[14]	0 (0)	0 (0)	0 (0)	4 (28.57)
Pakistan (775)	56 (7.23)	56 (7.23)	[14,36]	0 (0)	0 (0)	0 (0)	34 (30.36)
Total (881)	63 (7.15)	63 (7.15)		0 (0)	0 (0)	0 (0)	38 (30.16)
European							
French (100)	40 (40)	24 (24)	[10]	0 (0)	0 (0)	0 (0)	0 (0)
UK (142)	11 (7.75)	5 (3.52)	[50]	0 (0)	0 (0)	2 (20)	0 (0)
Czech (303)	26 (8.58)	8 (2.64)	[51]	0 (0)	0 (0)	0 (0)	0 (0)
Spanish (67)	19 (28.36)	18 (26.87)	[52]	0 (0)	0 (0)	2 (5.56)	0 (0)
Total (612)	96 (15.69)	55 (8.99)		0 (0)	0 (0)	4 (3.64)	0 (0)

**Table 6:** Correlation analysis of *SLC26A4* allele frequencies in the Mongolian cohort with multi-ethnic cohorts carrying biallelic *SLC26A4* variants.

Mongolian Variants	Mongolian (N = 15)	EA (N = 822)	NA (N = 66)	NEA (N = 104)	WA (N = 91)	SA (N = 63)	EUP (N = 55)
c.919-2A>G	0.400 (12/30)	0.6843 (1125/1644)	0.6667 (88/132)	0.1683 (35/208)	0.0659 (12/182)	0	0
c.2027T>A (p.L676Q)	0.233 (7/30)	0.0152 (25/1644)	0.1894 (25/132)	0.0096 (2/208)	0.0110 (2/182)	0	0
c.1318A>T (p.K440X)	0.167 (5/30)	0.0018 (3/1644)	0	0	0	0	0
c.1229C>T (p.T410M)	0.033 (1/30)	0.0249 (41/1644)	0	0.0240 (5/208)	0.0220 (4/182)	0	0.0364 (4/110)
c.281C>T (p.T94I)	0.033 (1/30)	0.0055 (9/1644)	0	0.0048 (1/208)	0	0	0
c.716T>A (p.V239D)	0.033 (1/30)	0.0006 (1/1644)	0	0	0.0330 (6/182)	0.3016 (38/126)	0
c.1547dup (p.S517FfsX10)	0.033 (1/30)	0.0030 (5/1644)	0	0	0	0	0
c.1975G>C (p.V659L)	0.033 (1/30)	0.0097 (16/1644)	0	0.0144 (3/208)	0	0	0
c.2089+1G>A	0.033 (1/30)	0.0012 (2/1644)	0	0	0	0	0
Pearson *r* [95% CI]		0.828 [0.368,0.963]	0.920 [0.657,0.983]	0.811 [0.318,0.959]	0.677 [0.023,0.925]	−0.222 [−0.772,0.518]	−0.222 [−0.772,0.518]
*p*-value		0.006	< 0.001	0.008	0.045	0.565	0.565

Abbreviations: EA, East Asian; NA, North Asian; NEA, Northeast Asian; WA, Western Asian; SA, South Asian; EUP, European.

## Data Availability

The datasets used and/or analyzed during the current study are available from the corresponding authors on reasonable request.

## Supplementary Materials

The following supporting information can be downloaded at: https://www.mdpi.com/article/10.3390/ijms27125364/s1.

## Abbreviations

EVA	Enlarged vestibular aqueduct
SNHL	Sensorineural hearing loss
IP-II	Partition type II
WES	Whole-exome sequencing
