# Supplementary material for: A Unique SLC26A4 Mutation Spectrum in a Mongolian Enlarged Vestibular Aqueduct Cohort via Whole-Exome Sequencing: A Preliminary Study
Source: Int J Mol Sci. 2026 Jun 14;27(12):5364. doi: 10.3390/ijms27125364 (PMC13300661; doi:10.3390/ijms27125364)
Supplement: Supplementary file 1 [file ijms-27-05364-s001.zip › ijms-4264047-supplementary.pdf]

International Journal of *Molecular Sciences*

MDPI

Supplementary Material

A Unique *SLC26A4* Mutation Spectrum in a Mongolian Enlarged Vestibular Aqueduct Cohort via Whole-Exome Sequencing: A Preliminary Study

Jargalkhuu Erdenechuluun [1,2,†], Bayasgalan Gombojav [3,†], Tserendulam Batsaikhan [2,4], Yue-Sheng Lu [5,6], Narandalai Danshiitsoodol [7], Zaya Makhbal [2,4], Maralgoo Jargalmaa [2,4], Tuvshinbayar Jargalkhuu [2,4], Ho-Peng Hsu [8], Pei-Hsuan Lin [5,9], Hung-Ju Su [10], Chien-Hsing Lin [10], Yu-Ting Chiang [11], Chuan-Jen Hsu [5,12], Pei-Lung Chen [6,9,11,13], Jacob Shu-Jui Hsu [11], Cheng-Yu Tsai [8,*] and Chen-Chi Wu [5,8,9,14,15,*]

[1] Department of Otolaryngology, School of Medicine, Mongolian National University of Medical Sciences, Ulaanbaatar 14210, Mongolia; jargalkhuu@mnums.edu.mn
[2] The EMJJ Otolaryngology Hospital, Ulaanbaatar 14210, Mongolia; tsek2036@gmail.com (T.B.); zaya.m@emjj.mn (Z.M.); jmaralgoo0313@gmail.com (M.J.); j.tuvshinbayar.t@gmail.com (T.J.)
[3] Department of Epidemiology and Biostatistics, School of Public Health, Mongolian National University of Medical Sciences, Ulaanbaatar 14210, Mongolia; bayasgalan.g@mnums.edu.mn
[4] International Cyber Education Center, Graduate School, Mongolian National University of Medical Sciences, Ulaanbaatar 14210, Mongolia
[5] Department of Otolaryngology, National Taiwan University Hospital, Taipei 100225, Taiwan; g05092lu@gmail.com (Y.-S.L.); peihsuanlin@ntu.edu.tw (P.-H.L.); cjhsu@ntu.edu.tw (C.-J.H.)
[6] Institute of Molecular Medicine, National Taiwan University College of Medicine, Taipei 100225, Taiwan; paylong@ntu.edu.tw
[7] Department of Probiotic Sciences for Preventive Medicine, Graduate School of Biomedical and Health Sciences, Hiroshima University, Hiroshima 734-8553, Japan; naraa@hiroshima-u.ac.jp
[8] Department of Otolaryngology, National Taiwan University College of Medicine, Taipei 100225, Taiwan; pondyhsu920@gmail.com
[9] Graduate Institute of Clinical Medicine, National Taiwan University College of Medicine, Taipei 100229, Taiwan
[10] Phalanx Biotech Inc., Hsinchu 302401, Taiwan; richardsu@phalanxbiotech.com (H.-J.S.); jameslin@phalanxbiotech.com (C.-H.L.)
[11] Graduate Institute of Medical Genomics and Proteomics, National Taiwan University College of Medicine, Taipei 100233, Taiwan; ashley.chiang413@gmail.com (Y.-T.C.); jacobhsu@ntu.edu.tw (J.S.-J.H.)
[12] Department of Otolaryngology, Taichung Tzu Chi Hospital, Taichung 427003, Taiwan
[13] Department of Medical Genetics, National Taiwan University Hospital, Taipei 100226, Taiwan
[14] Department of Medical Research, National Taiwan University Hospital Hsin-Chu Branch, Hsinchu 302041, Taiwan
[15] Department of Otolaryngology, National Taiwan University Hospital Hsin-Chu Branch, Hsinchu 302041, Taiwan
* Correspondence: leontsai9139@ntu.edu.tw (C.-Y.T.); chenchiwu@ntuh.gov.tw (C.-C.W.)
† These authors contributed equally to this work.

 https://doi.org/10.3390/ijms27125364

International Journal of *Molecular Sciences*

MDPI

Table S1. Summary of case ratios and allelic frequencies of Mongolian *SLC26A4* variants in bi-allelic genotype across multi-ethnic deaf cohorts

**Population**	**Ratios of samples with any genotype of *SLC26A4* variants**	**Ratios of samples with *SLC26A4* biallelic variants**	**Ref**	**The allelic frequency of each Mongolian *SLC26A4* variant in populations with biallelic genotype**								
				**c.919-2A>G**	**c.2027T>A (p.L676Q)**	**c.1318A>T (p.K440X)**	**c.1229C>T (p.T410M)**	**c.281C>T (p.T94I)**	**c.716T>A (p.V239D)**	**c.1546dupC (p.S517FfsX10)**	**c.1975G>C (p.V659L)**	**c.2089+1G>A**
**East Asian**												
**Taiwanese (N=5184)**	**346 / 5184 (6.67%)**	300 / 5184 (5.79%)	[32]	473 / 600 (78.83%)	0 / 600 (0%)	2 / 600 (0.33%)	17 / 600 (2.83%)	0 / 600 (0%)	0 / 600 (0%)	0 / 600 (0%)	1 / 600 (0.17%)	0 / 600 (0%)
Chinese (N=3379)	654 / 3379 (19.35%)	522/ 3379 (15.45%)	[41, 42]	652 / 1044 (62.45%)	25 / 1044 (2.39%)	1 / 1044 (0.1%)	24 / 1044 (2.3%)	9 / 1044 (0.86%)	1 / 1044 (0.1%)	5 / 1044 (0.48%)	15 / 1044 (1.44%)	2 / 1044 (0.19%)
Total (N=8563)	1000 / 8563 (11.68%)	822 / 8563 (9.6%)		1125 / 1644 (68.43%)	25 / 1644 (1.52%)	3 / 1644 (0.18%)	41 / 1644 (2.49%)	9 / 1644 (0.55%)	1 / 1644 (0.06%)	5 / 1644 (0.3%)	16 / 1644 (0.97%)	2 / 1644 (0.12%)
North Asian												
Tuvinian (N=220)	75 / 220 (34.09%)	62 / 220 (28.20%)	[43]	86 / 124 (69.35%)	23 / 124 (18.55%)	0 / 124 (0%)	0 / 124 (0%)	0 / 124 (0%)	0 / 124 (0%)	0 / 124 (0%)	0 / 124 (0%)	0 / 124 (0%)
Altaian (N=93)	5 / 93 (5.38%)	4 / 93 (4.30%)	[43]	2 / 8 (25%)	2 / 8 (25%)	0 / 8 (0%)	0 / 8 (0%)	0 / 8 (0%)	0 / 8 (0%)	0 / 8 (0%)	0 / 8 (0%)	0 / 8 (0%)
Total (N=313)	80 / 313 (25.56%)	66 / 313 (21.09%)		88 / 132 (66.67%)	25 / 132 (18.94%)	0 / 132 (0%)	0 / 132 (0%)	0 / 132 (0%)	0 / 132 (0%)	0 / 132 (0%)	0 / 132 (0%)	0 / 132 (0%)
Northeast Asian												
Japanese (N=1511)	100 / 1511 (6.62%)	66 / 1511 (4.37%)	[44]	13 / 132 (9.85%)	0 / 132 (0%)	0 / 132 (0%)	3 / 132 (2.27%)	1 / 132 (0.48%)	0 / 132 (0%)	0 / 132 (0%)	3 / 132 (2.27%)	0 / 132 (0%)
Korean (N=323)	63 / 323 (19.50%)	38 / 323 (11.76%)	[14, 45]	22 / 76 (28.95%)	2 / 76 (2.63%)	0 / 76 (0%)	2 / 76 (2.63%)	0 / 76 (0%)	0 / 76 (0%)	0 / 76 (0%)	0 / 76 (0%)	0 / 76 (0%)
Total (N=1834)	163 / 1834 (8.89%)	104 / 1834 (5.67%)		35 / 208 (16.83%)	2 / 208 (0.96%)	0 / 208 (0%)	5 / 208 (2.40%)	1 / 208 (0.48%)	0 / 208 (0%)	0 / 208 (0%)	3 / 208 (1.44%)	0 / 208 (0%)
Western Asian												
Iranian (N=831)	76 / 831 (9.15%)	74 / 831 (8.9%)	[46]	8 / 148 (5.41%)	2 / 148 (1.35%)	0 / 148 (0%)	2 / 148 (1.35%)	0 / 148 (0%)	6 / 148 (4.05%)	0 / 148 (0%)	0 / 148 (0%)	0 / 148 (0%)
Turkey	17 / 133	17 / 133	[47-49]	4 / 34	0 / 34	0 / 34	2 / 34	0 / 34	0 / 34	0 / 34	0 / 34	0 / 34

https://doi.org/10.3390/ijms27125364

(N=133)	(12.78%)	(12.78%)		(11.76%)	(0%)	(0%)	(5.88%)	(0%)	(0%)	(0%)	(0%)	(0%)
Total	93 / 964	91 / 964		12 / 182	2 / 182	0 / 182	4 / 182	0 / 182	6 / 182	0 / 182	0 / 182	0 / 182
(N=964)	(9.65%)	(9.44%)		(6.59%)	(1.10%)	(0%)	(2.20%)	(0%)	(3.30%)	(0%)	(0%)	(0%)
South Asian												
Indian	7 / 106	7 / 106	[14]	0 / 14	0 / 14	0 / 14	0 / 14	0 / 14	4 / 14	0 / 14	0 / 14	0 / 14
(N=106)	(6.60%)	(6.60%)		(0 %)	(0 %)	(0 %)	(0 %)	(0 %)	(28.57%)	(0 %)	(0 %)	(0 %)
Pakistan	56 / 775	56 / 775	[14, 36]	0 / 112	0 / 112	0 / 112	0 / 112	0 / 112	34 / 112	0 / 112	0 / 112	0 / 112
(N=775)	(7.23%)	(7.23%)		(0 %)	(0 %)	(0 %)	(0 %)	(0 %)	(30.36%)	(0 %)	(0 %)	(0 %)
Total	63 / 881	63 / 881		0 / 126	0 / 126	0 / 126	0 / 126	0 / 126	38 / 126	0 / 126	0 / 126	0 / 126
(N=881)	(7.15%)	(7.15%)		(0%)	(0%)	(0%)	(0%)	(0%)	(30.16%)	(0%)	(0%)	(0%)
European												
French	40 / 100	24 / 100	[10]	0 / 48	0 / 48	0 / 48	0 / 48	0 / 48	0 / 48	0 / 48	0 / 48	0 / 48
(N=100)	(40.00%)	(24.00%)		(0%)	(0%)	(0%)	(0%)	(0%)	(0%)	(0%)	(0%)	(0%)
UK	11 / 142	5 / 142	[50]	0 / 10	0 / 10	0 / 10	2 / 10	0 / 10	0 / 10	0 / 10	0 / 10	0 / 10
(N=142)	(7.75%)	(3.52%)		(0%)	(0%)	(0%)	(20.00%)	(0%)	(0%)	(0%)	(0%)	(0%)
Czech	26 / 303	8 / 303	[51]	0 / 16	0 / 16	0 / 16	0 / 16	0 / 16	0 / 16	0 / 16	0 / 16	0 / 16
(N=303)	(8.58%)	(2.64%)		(0%)	(0%)	(0%)	(0%)	(0%)	(0%)	(0%)	(0%)	(0%)
Spanish	19 / 67	18 / 67	[52]	0 / 36	0 / 36	0 / 36	2 / 36	0 / 36	0 / 36	0 / 36	0 / 36	0 / 36
(N=67)	(28.36%)	(26.87%)		(0%)	(0%)	(0%)	(5.56%)	(0%)	(0%)	(0%)	(0%)	(0%)
Total	96 / 612	55 / 612		0 / 110	0 / 110	0 / 110	4 / 110	0 / 110	0 / 110	0 / 110	0 / 110	0 / 110
(N=612)	(15.69%)	(8.99%)		(0%)	(0%)	(0%)	(3.64%)	(0%)	(0%)	(0%)	(0%)	(0%)

International Journal of
Molecular Sciences

MDPI

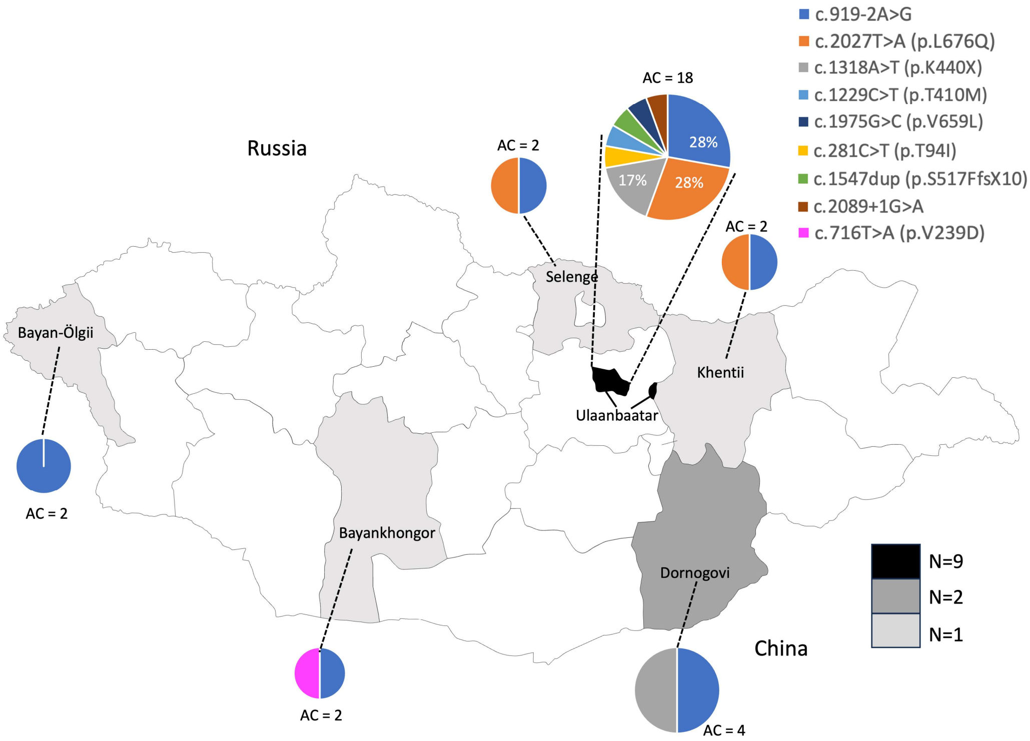

Figure S1. Geographic distribution of birthplace statistics and pathogenic variants of *SLC26A4*-positive Mongolian patients enrolled in this study. Abbreviation: AC, Allelic counts (on unrelated families).

 https://doi.org/10.3390/ijms27125364
